# Assessment and Remediation of Heavy Metal Contamination in Soil

**DOI:** 10.3390/toxics14060526

**Published:** 2026-06-17

**Authors:** Yangyang Wang

**Affiliations:** 1 Faculty of Geographical Science and Engineering, College of Geographical Science, Henan University, Zhengzhou 450046, China; wangyangyangxyz@163.com; Tel.: +86-15194602157; 2 Key Laboratory of Geospatial Technology for the Middle and Lower Yellow River Regions (Henan University), Ministry of Education, Kaifeng 475004, China

Anthropogenic activities, including industrial production, mineral exploitation, metal smelting, agricultural fertilizer and pesticide application, as well as livestock and poultry breeding, have released large amounts of heavy metals into the environment [1]. These heavy metals can enter the soil through various pathways, ultimately causing severe soil heavy metal contamination [2]. Due to the persistence and irreversibility of heavy metal contamination, they can be biomagnified through the food chain and accumulate in the human body. Heavy metals accumulation in humans can damage the nervous system, liver and kidney metabolism, and immune system, induce multiple chronic diseases, and seriously threaten human health [3].

In-depth research on the monitoring, risk assessment, and remediation technologies for soil heavy metal contamination has been conducted over several decades [4], and traditional pollution monitoring methods, risk evaluation models, and remediation technologies have become relatively sophisticated [5]. Accordingly, the research focus of a large number of researchers has gradually shifted towards the identification, risk evaluation, and remediation of emerging pollutants [6,7], which weakens the remediation research of heavy metal contamination in soil to some extent. Even so, heavy metal contamination in soil still widely persists. It is worth noting that the rapid development of emerging technologies (big data and artificial intelligence) has provided new ideas and technical support for the precise traceability, quantitative risk assessment, and efficient remediation of heavy metal contamination [8]. Based on this, in-depth research on the risk assessment and precise remediation of heavy metal contamination can effectively solve the problem of regional heavy metal contamination, which is of great significance for maintaining regional ecological environment stability, safeguarding human health, and promoting the high-quality development of the ecological environment.

This Special Issue collects 12 research papers focusing on the hazards, potential risks, and remediation techniques of soil heavy metal contamination. Among these works, Mi et al. (Contribution 1) adopted high-throughput sequencing to explore how long-term heavy metal contamination alters soil physicochemical properties as well as the composition and diversity of soil bacterial and fungal communities. Their research results showed that long-term heavy metal contamination can seriously affect the beta diversity of soil bacteria and fungi, and the soil community structure was significantly changed. The bacterial community was mainly regulated by soil available phosphorus, available Cd, and available Pb, while the fungal community was mainly controlled by soil available phosphorus, soil organic carbon, and total Pb content (Contribution 1). In addition, other studies have analyzed the heavy metals pollution status in various environmental media in typical regions. For example, Zhang et al. (Contribution 2) analyzed the contents and potential risks of heavy metals in soils and sediments around the Zoige Uranium Mine; He et al. (Contribution 3) studied the potential risks and sources of heavy metals in farmland soils in the Nanyang Basin; Jiang et al. (Contributions 4 and 5) analyzed the pollution status and risks of heavy metals in the Huang-Huai-Hai Plain and paddy fields; Liu et al. (Contribution 6) analyzed the long-term trends and potential risks of heavy metals in farmland soil in the Songnen Plain; Wang et al. (Contribution 7) even conducted a detailed analysis on the contents and risks of heavy metals in the surface dust from underground parking garages. These studies have provided detailed insights into the pollution status and potential risks of heavy metals across various typical regions, providing data support for subsequent heavy metal contamination control and remediation.

In addition, several studies have also explored the remediation efficiency of various technologies for soil heavy metal contamination. Herrera Figueroa et al. (Contribution 8) investigated differential accumulation features of different heavy metal in sorghum and clarified pollution sources in sorghum grains, verifying its feasibility for remediating heavy metals-contaminated soil. Yusuyin et al. (Contribution 9) analyzed the Cd accumulation characteristics in 16 woody plants and screened candidates for Cd phytoremediation, supplying available plants for Cd-contaminated soil remediation. Moreover, numerous studies have also evaluated the stabilization performance of multiple amendments to soil heavy metals. Chen et al. (Contribution 10) explored the remediation potential of N-modified biochar for severely heavy metal contaminated soil. The N-modified biochar reduced the heavy metal bioavailability in soil and inhibited heavy metal uptake by Chinese cabbage to a certain extent, but the heavy metal concentrations in spinach still exceeded China’s standard limit [9]. Wang et al. (Contribution 11) examined synergistic stabilization of Sb and As in soil by iron–manganese-modified hydrochar. Their findings revealed that 5% (*w*/*w*) hydrochar application enables synchronous and durable stabilization of arsenic and antimony in soil. Weng et al. (Contribution 12) synthesized zirconia nanoparticles with *Sonchus asper* extract for chromium-contaminated soil remediation, and the prepared nanoparticles improved Chinese cabbage growth while suppressing its chromium accumulation. Collectively, these studies provide optional approaches for phytoremediation and in situ stabilization of heavy metals-contaminated soils.

Although significant progress has been made in the remediation of heavy metal contamination in soil, we must be aware that extensive studies are still needed for the effective remediation of heavy metal-contaminated soil. In the future, studies can be conducted in the following areas: (1) big data and artificial intelligence can provide new ideas for the remediation of soil heavy metal contamination [10,11], which can greatly improve the accuracy and efficiency of soil heavy metal contamination assessment and remediation; (2) with the rapid development of material technology, the development of innovative high-performance functional materials can provide more possibilities for the remediation of soil heavy metal contamination [12,13]; (3) microorganisms have demonstrated strong remediation capabilities to various pollutants [14,15], and the rapid development of biotechnology can provide more alternative solutions for heavy metal contamination; (4) although various technologies can remediate soil heavy metal contamination to some extent, attention must be paid to the potential impacts of different remediation techniques on the soil environment and secondary pollution [16]. These studies can further improve the technology system for soil heavy metal remediation and achieve safe utilization of heavy metal-contaminated soil.

This Special Issue mainly presents the latest research advances in heavy metal pollution assessment and remediation. We thank all authors who contributed to this Special Issue.

## Data Availability

Not applicable.
